# Fabrication of Large-Area High-Resolution Templates by Focused Ion Beam Combined with Colloidal Nanoparticle Dimer Deposition for SERS Substrates

**DOI:** 10.3390/nano14221784

**Published:** 2024-11-06

**Authors:** Liga Ignatane, Reinis Ignatans, Juris Prikulis, Annamarija Trausa, Ciro Federico Tipaldi, Edgars Vanags, Martins Zubkins, Krisjanis Smits, Anatolijs Sarakovskis

**Affiliations:** 1 Institute of Solid State Physics, University of Latvia, 8 Kengaraga St., LV-1063 Riga, Latvia; 2 Institute of Chemical Physics, Faculty of Science and Technology, University of Latvia, 1 Jelgavas St., LV-1004 Riga, Latvia

**Keywords:** focused ion beam, nanostructure fabrication, dimers, SERS, plasmonics

## Abstract

This article presents an examination of well-controlled patterns created using a Ga^+^-based focused ion beam (FIB) on glass, while silicon substrates were used to evaluate the FIB performance by its achievable feature size versus time constraints. The pattern creation on glass was developed with the aim of studying potential surface-enhanced Raman spectroscopy (SERS) applications. Furthermore, the FIB was used to create dimer systems of periodically and randomly positioned dumbbell-shaped pits on the glass (each dimer occupies an area of 203 × 87 nm^2^). By following the bitmap pattern files, the FIB ensured there was 3000 dimer fabrication over a 20 × 20 μm^2^ large area, with a pit size and position variation below 10 nm. The article highlights that FIB can be used for precise large-area nano-fabrication. The gold nanoparticle dimers were formed on the prepatterned surface via capillary force-assisted deposition. The fabricated nanostructures were tested in SERS measurements. The enhancement factor for Rhodamine B molecule reached ~10^5^, demonstrating the potential application of the method to create nanostructures in the sensor domain.

## 1. Introduction

Raman scattering originates from electromagnetic radiation inelastic scattering due to photon–phonon/molecule vibration coupling. However, the conventional Raman spectroscopy signal is weak, which hinders the detection of molecules at low concentrations. The discovery of Raman signal enhancement by a rough silver layer led to the development of a technique known as SERS, meaning surface-enhanced Raman spectroscopy [1,2].

Silver is considered to be one of the best, or even the best, metal for SERS applications, due to its excellent surface plasmon polariton (SPP) and local surface plasmon resonance (LSPR) quality factor in the visible and near-infrared (NIR) range [3]. However, silver has a major drawback, as it easily oxidizes and degrades, especially if particles are nanosized. As an alternative to silver, gold can be used to form SERS-active sites. Although the enhancement properties of gold-based nanostructures are not as good as silver-based ones, their chemical stability and biocompatibility [4,5] enable their use as SERS biosensors in living organisms.

The electric field component of surface plasmon modes decreases exponentially by increasing the distance from the surface [6]; therefore, molecules adsorbed at a nanometric distance experience a stronger electric field, contributing to a stronger Raman signal. Furthermore, the exact geometry of the nanosized object plays a role in shaping the electric field distribution. In recent decades, countless types of SERS substrates have been employed to increase the surface area for adsorbents and create electromagnetic “hot spots”. These include nanoparticles (NPs) [7,8], bowtie antennas [9], nanorings [10], nanovoids [11], nanopillars [12], and dimer systems [13,14]. Additionally, if a “hot spot” is engineered to attract molecules of interest, it would create an excellent SERS substrate, with tailored selectivity [15]. However, an effective SERS substrate analysis requires the fabrication of precise, repeatable nanostructures, as their size, shape, and positioning significantly influence Raman signal enhancement, necessitating high reproducibility for consistent results [1].

Spherical nanoparticle dimer systems are particularly interesting for fundamental insights into the interactions between NPs in close proximity and plasmonic effects [16]. The interaction between two metallic nanoparticles generates coupled LSPR, enabling significant local field enhancement, potentially reaching the value of 10^9^–10^11^ [17]. The SERS enhancement factor at these hot spots is highly sensitive to the particle gap; even a 1 nm change can alter the enhancement by an order of magnitude [18]. The distance control between NPs can be achieved via chemical bond engineering [16,19,20], or by precisely fabricating holes in the substrate, where the NPs get stuck at predetermined positions [21,22,23]. Similarly, NPs are fixed on the substrate either by a chemical bond or by physical holes in the substrate. The chemical fixation route offers extraordinary precision of the distance control between the dimers’ particles [16], but poor positional control of the particles on the substrate (i.e., they are positioned rather randomly). The opposite is true for the physically fabricated substrates, wherein there is great positional control on the substrate [23], but weaker distance control of the nanoparticles’ dimer.

Modern lithography methods demonstrate repeatable fabrication processes. Even more, these methods have the ability to control and modify all three parameters (size, shape, and position) to test possible surface solutions for SERS. The wavelength of light limits conventional photolithography (visible/near UV); thus, fabricated feature sizes start from ~1 μm. Electron beam lithography (EBL) can achieve a sub-10 nm resolution; therefore, it is more suitable for nanostructure fabrication. Still, EBL has limitations in the selection of substrates, as the process is not direct (specialized masks are used, and additional etching/deposition processes are used outside of the machine), and, if compared to conventional photolithography, it is more expensive.

Between those lithography methods, there is a technique mostly used for transmission electron microscopy (TEM) lamellae preparation, which is called FIB. FIB interacts directly with the substrate without any additional post-processing (i.e., removal of the photoresist, wet chemical etching). At the lower beam currents (~1 pA) for Ga^+^ FIB, the minimum size of the structure in a bulk material is 9 nm [24], and a smaller feature size (approximately 3.5 nm) can be achieved by patterning free-standing graphene [25]. In both cases, the feature size is comparable to the EBL resolution.

Nowadays, many scanning electron microscopes (SEM) are equipped with FIB; thus, its accessibility makes it more attractive than EBL. With proper preconditions, FIB can be used on almost any solid substrate, which is a huge advantage. Fabrication using FIB is flexible, as it is easy to change the area of interest, etching area size, and depth, even on the same surface. Thus, it could be applied for fabricating nanostructures that could be tested as SERS substrates.

Production of nanostructured surfaces with controlled periodicity and disorder is of great interest for the development of optical applications, including imaging [26], photocatalysis [27], surface plasmon sensors [28], or substrates for surface-enhanced Raman spectroscopy SERS [29]. While regular metal nanoparticle arrays can produce spectral peaks with a high-quality factor, due to plasmonic surface lattice resonance [30], disordered arrays result in the formation of local high-intensity regions, or “hot spots”, upon excitation with light at frequencies that are close to the individual particle resonance [31].

Traditionally, pits for NPs are fabricated on silicon wafer substrates using various etching methods [21,22,23]. In this article, we emphasize the FIB capability to fabricate high-resolution nanostructures in a large area, specifically demonstrating the application of FIB for the fabrication of SERS substrates on glass. A glass substrate allows for additional optical characterization and optical sensing possibilities. Even on an optical fiber, the plasmonic structures can be integrated with FIB, despite its dielectric nature and unhandy shape [32]. Additionally, we employ the possibility of precise positional control of the substrate fabrication to compare the effects of the dimer pattern angular distribution, which is as follows: randomly positioned dimers versus dimers positioned periodically in one direction.

The aim of this work was to create nanopatterned glass substrates with FIB that could be used as a proof of concept for SERS studies. The following three tasks were set: (1) an experimental study of FIB Ga^+^ fabrication accuracy; (2) automating the FIB fabrication process to pattern large areas with homogenous dimer pits; and (3) creating two different designs of SERS substrates on glass.

## 2. Materials and Methods

### 2.1. Materials

Prior to surface-enhanced Raman scattering substrate fabrication, focused ion beam resolution, accuracy, and precision tests were made on a conventional silicon wafer.

A glass slide was chosen as a substrate for FIB patterning. It was divided by a diamond pen into 12 × 9 mm large pieces. Afterwards, the glass was cleaned in an ultrasonic bath three times (first in deionized water, then in acetone, and finally in isopropanol), each time for 15 min. The cleaned glass was then broken into rectangles.

Before surface fabrication by FIB, a 25 nm silver film was deposited on the soda-lime glass substrate using the R&D multifunctional vacuum coater SAF25/50 from Sidrabe Vacuum, Ltd., to minimize glass charging effects during electron and ion interactions. The deposition rate was 1 Å/s, at a pressure of 0.7 mPa.

After FIB fabrication, the silver film was chemically removed by immersing the substrate into 70% nitric acid. The remaining acidic residues were rinsed off with deionized water.

Au NPs with a 60 nm diameter stabilized colloidal suspension in 0.1 mM phosphate-buffered saline (PBS) (Sigma-Aldrich 753653, Saint Louis, MO, USA) were deposited on the FIB-patterned templates using convective capillary force-assisted deposition [33]. The substrates were vertically immersed in the colloid at room temperature and atmospheric pressure, then withdrawn at a velocity of 0.1 µm/s using a custom-designed dip-coating apparatus. This allowed for the trapping of NPs in the dumbbell-shaped pits, either as monomers or dimers. After withdrawal from the colloidal solution (overnight), the samples were left to dry in ambient air for several hours. The samples were subsequently rinsed in water in order to dissolve PBS residues, while the trapped NPs remained in the pits. The sample stability is ensured by careful matching of the NP radius and pit curvature, which maximizes the NP–substrate contact area and van der Waals adhesion force [34]. Once trapped, the NPs remain in the pits and withstand extended rinsing and sonication.

The Rhodamine B (RhB) solution in isopropanol was chosen as the SERS detection dye. RhB (dye content ≥90%) and isopropanol (ACS reagent, ≥99.5%) were purchased from Sigma-Aldrich. A 6.1 × 10^−5^ M RhB solution in isopropanol was prepared by dissolving RhB in isopropanol to achieve the desired concentration.

### 2.2. Methods

The nanostructure fabrication and visualization were obtained by using the dual-beam microscope Helios 5 UX (Thermo Fisher Scientific, Eindhoven, The Netherlands). Dumbbell-shaped pits were fabricated using the Phoenix Gallium Ion Column, at an ion beam voltage of 30 kV and current of 7 pA. Fabricated structures were visualized using an ultra-high-resolution Elstar electron column with the UC+ source mode at 5 kV and 0.10 nA beam current, with a Through Lens Detector (TDL).

A transmission electron microscope FEI Tecnai at 200 kV was used to visualize cross-sections of nanostructures.

Dark-field (DF) spectra were collected using a fiber-coupled spectrometer (Andor Technology, Belfast, Northern Ireland, SR-163, grating 600 lines/mm, 500 nm blaze, detector DV401A-BVF) connected to an inverted microscope (Olympus IX71, Tokyo, Japan), with a DF condenser lens (U-TLD, numerical aperture NA = 0.9) and a 60× objective lens (LUCPlanFLN NA = 0.7). A halogen lamp (Olympus U-LH100L-3) was used as a light source.

Raman spectroscopy was carried out using the Princeton Instruments TriVista CRS Confocal Raman Microscope (TR777), S&I Spectroscopy & Imaging GmbH, Warstein, Germany. The instrument is equipped with three monochromators and a microscope. The 600 lines/mm diffraction grating was used, and the Olympus Mplan N 50× objective with a numerical aperture of NA = 0.75 and a working distance of 0.38 mm was chosen. This configuration produces a laser spot size of about 2 μm. The slit aperture of the microscope was specifically adjusted to 200 μm. For excitation purposes, a Cobolt Samba 150 (HÜBNER Photonics GmbH, Kassel, Germany) 532 nm YAG diode laser with a power of 2.55 mW was used on the sample. Each area of the FIB-fabricated structures was measured at 3 points to achieve a more complete dataset of the spectra, characterizing the average performance of the substrate.

### 2.3. Software

Automation of the Thermo Scientific dual-beam microscope Helios 5 UX was conducted using iFast Developer’s Hit software Professional version 6.14.2.251. The bitmaps of dumbbell-shaped systems were generated by Mathematica; version 12.1; Wolfram Research, Inc.: Champaign, IL, USA. The scanning electron microscopy (SEM) image analyses were obtained using Fiji ImageJ [35].

## 3. Results

### 3.1. FIB Ga^+^ Fabrication Accuracy

**Figure 1:**
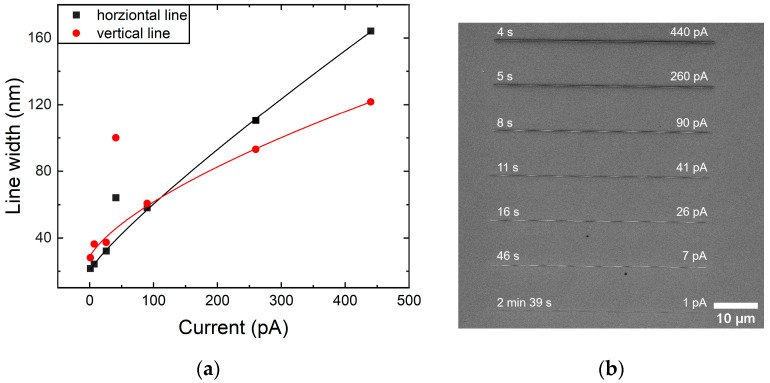
Ga^+^ ion etching of a one pixel wide, 50 μm long, and 100 nm deep line. (**a**) Graph of a line width depending on the FIB current at an accelerating voltage of 30 kV; the lines serve as eye guides, and points that fall out of trend correspond to a frequently used aperture for lamellae preparation, and (**b**) a SEM image of horizontally etched lines with added fabrication time on the left and the FIB current on the right.

To understand the limitations and choose the optimal strategy, the FIB etching resolution was checked on a silicon wafer. One pixel wide (corresponding to ~11 nm) lines with the same length and depth were etched at 30 kV with variable beam currents to test the process’ accuracy (Figure 1b). The obtained results show that the etched line width increases with a higher FIB current, as was expected (Figure 1a). A smaller current corresponds to a smaller aperture, which results in a finer FIB spot size on the surface, allowing for the fabrication of smaller structures. On the other hand, high-resolution etching has a slower material removal rate, drastically increasing the operational time. Therefore, the optimal beam current must be chosen to achieve the planned feature sizes, while at the same time keeping the patterning time reasonable. Consequently, to etch structures at or below ~100 nm, only 90 pA and less Ga^+^ ion currents can be used. The width of horizontally (along the scan direction) and vertically (perpendicular to the scan direction) etched lines were compared, as shown in Figure 1a. In the FIB etching of mutually perpendicular lines, there is a noticeable difference in the line width, which highlights the variability of the non-circular FIB probe shape and scan coil strength deviations, with respect to the etching direction. Furthermore, the etch rate varies not only among the materials, but also across different crystallographic planes of single crystals (e.g., Si wafer), which could also be the reason for the observed deviation of line widths.

**Figure 2:**
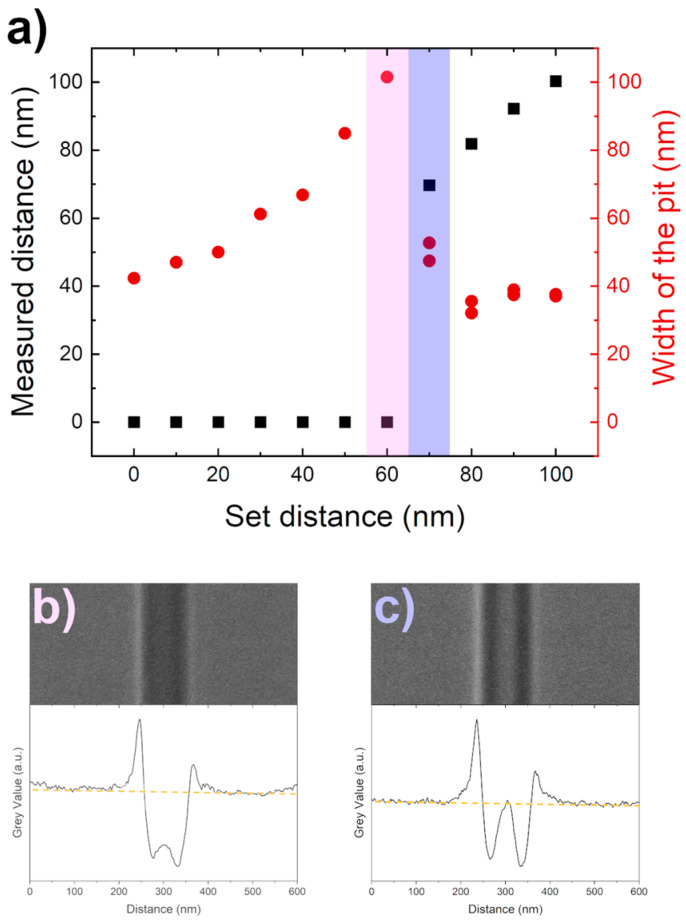
The FIB resolution tests at 30 kV and 7 pA. (**a**) Recognized distance between two lines (black squares) and the width of the line pit (red dots) depending on the set distance between two lines. SEM image and corresponding integrated gray value graph of (**b**) the biggest set distance when two lines cannot be distinguished (corresponding to the pink area in graph (**a**)), and (**c**) the smallest set distance when two lines can be distinguished (corresponding to the purple area in graph (**a**)); dashed yellow line in graphs (**b**,**c**) represents BG level.

The proposed FIB-fabricated dimer system consists of two pits connected with a small (12 nm) gap. For that purpose, several parallel lines were etched, increasing in the FIB the set distances between them to understand when two lines can be distinguished. The etched lines were analyzed by integrating pixel gray values of the SEM image with a 200 pixels wide line, crossing them perpendicularly (Figure 2b,c). The etched structure was recognized in regions where gray values were lower than the background (BG) level (Figure 2b). The etched structure was considered to consist of two objects if somewhere between the etched structures the gray value was the same or higher than the BG level (Figure 2c). Structure widths were measured from gray value graphs as the distance between two points, where the gray value is below the BG level. The etched structure width shows little variation, as the width of fully overlapping two lines is 42 nm, while it is 37–39 nm for completely separated lines. The observed broadening of width is caused by a decrease in the overlapping area of two lines until the separation point is reached (Figure 2a, red dots).

The measured distance between two distinct lines was recognized as the distance between the minimum gray value points in the graph. The set distance and measured (or recognized distance) fit well (Figure 2a black squares), which indicates appropriately tailored image analysis and well-controlled FIB etching. Two ~ 40 nm wide structures etched by FIB at 30 kV and 7 pA can be distinguished if they are set at least 70 nm apart (Figure 2a, highlighted in purple and expanded in Figure 2c). Such an observation highlights that the resolution of two FIB-etched structures depends on the smallest structure’s fabrication capability.

**Figure 3:**
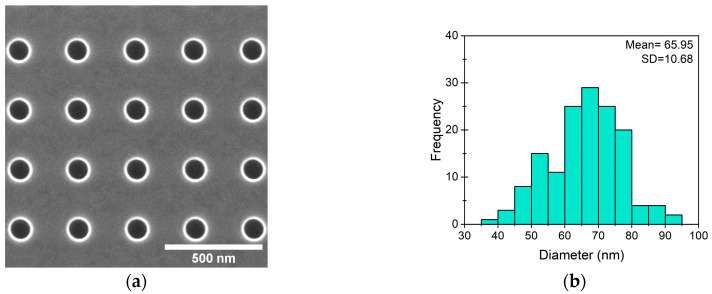

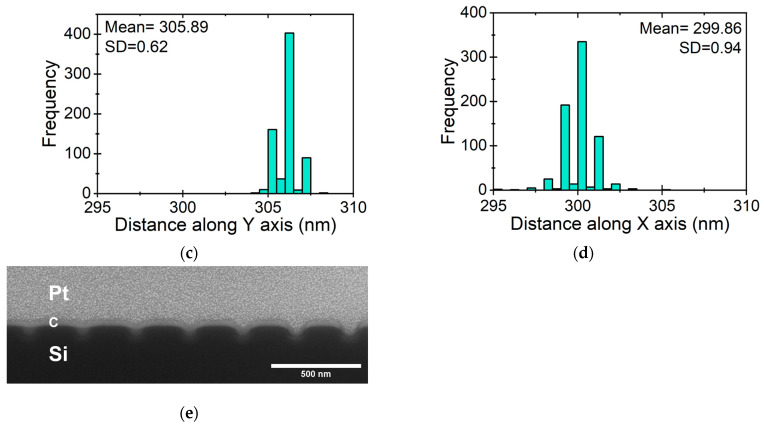
Characterization of periodically positioned pits. (**a**) SEM image of FIB-etched pits, set 100 nm in diameter, 100 nm in depth, and 300 nm apart from each other; (**b**) Diameter distribution of pits; (**c**) Distribution of distance between pits along *Y*-axis and (**d**) along *X*-axis; (**e**) SEM image of lamella consisting of pits’ cross-sections.

The planned SERS substrate consisted of precisely positioned pits created by FIB etching. For FIB-created pit characterization, pits of 100 nm in diameter, with 100 nm depth, and a period 300 nm apart were set to be etched by FIB on the silicon wafer (Figure 3a). The mean diameter of the fabricated pits was 105.5 nm, measured from SEM images, which is slightly larger than the set diameter (Figure 3b). The periodicity was tested by finding the mass center for each pit using ImageJ in SEM image, and then measuring the distance between them along the perpendicular axis. The set periodicity for etching was 300 nm, but the measured distance between etched pits was 300 nm along the *X*-axis and 306 nm along the *Y*-axis (Figure 3c,d). The observed difference in *Y*-axis periodicity is a 2% error from the set distance, which most likely is a result of a slight shift of the coincident angle of the FIB sample. Possible origins of these deviations are as follows: instrumental (the sample holder and FIB column angle matching problems) or the plane of the substrate surface is not completely parallel to the surface of the sample holder.

**Figure 4:**
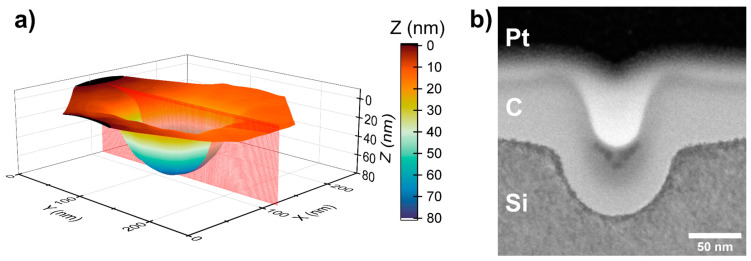
Characterization of a pit fabricated by FIB. (**a**) 3D visualization of the pit with a set diameter of 100 nm and a depth of 100 nm, created from measured cross-sectional planes, wherein the red plane corresponds to image (**b**); (**b**) TEM image of pit’s cross-section.

The depth of the pits was characterized by cutting out a lamella from the substrate consisting of fabricated pits. Before the lamella preparation, the surface from further ion interactions was protected by coating it with electron-deposited carbon, which was followed by ion-deposited platinum. The lamella was polished at a small angle, with respect to the row of pits, so that the pits could be seen in various cross-sectional planes (Figure 3e). Using ImageJ, the TEM images of the lamella were analyzed [25], and the x and y coordinates were registered along the Si/C contact line and fitted with each other by taking into account the ion-“untouched” Si plane and pits’ maximum (or the deepest point) position (Figure 4b). A 3D visualization was done by stitching together such cross-sectional planes and using symmetric functions (Figure 4a). The ion-“untouched” Si surface was defined as a 0 nm depth point, and the pit depth was measured from it. The pit reconstruction revealed that they are around 80 nm deep. The described nanostructure cross-sectional characterization has its limitations, as the precision of the lamella’s final polishing with gallium FIB is comparable to its nanostructure size. However, the results demonstrate the characteristic geometry of FIB-fabricated pits and give an understanding of the set etch depth for the planned pit, considering the substrate material.

### 3.2. Automating the FIB Fabrication Process to Pattern Large Areas with Homogenous Dimer Pits

**Figure 5:**
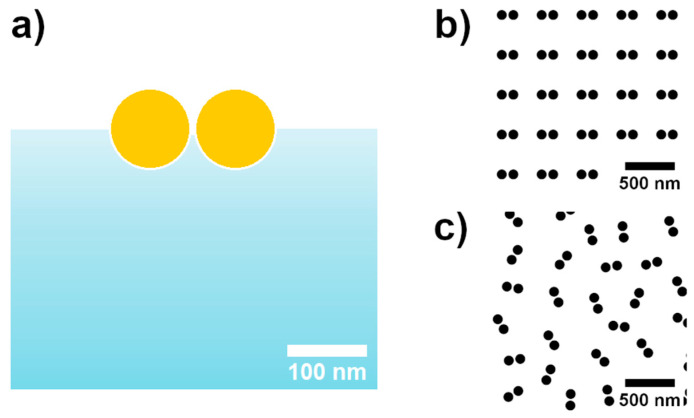
Schematic of the SERS substrate element (**a**) cross-section model of the envisioned dimer system and bitmaps of (**b**) periodically positioned and (**c**) randomly positioned dimer systems as a pattern for FIB fabrication.

We planned to create a pattern of dimer systems (dumbbell-shaped pits) on glass filled with two gold sphere-like NPs that were close to each other, but not in contact (Figure 5a). The pit preparation approach was used to study two dimer systems, as follows: periodically positioned (Figure 5b) and randomly positioned dimers (Figure 5c). Technically, it is ensured by dimer pits fabricated with FIB on the glass covered with a 25 nm Ag layer (used to prevent charging during fabrication process). Further, the Ag layer was chemically etched away with 70% HNO_3_, and 60 nm diameter gold NPs from the colloidal solution were filled into the pits using a capillary force-assisted deposition technique [33]. The smaller gold NPs corresponding to pit diameter were chosen to increase NPs insertion into the pits [22]. FIB exposure is controlled by previously prepared pattern bitmap files, where pixel RGB defines whether pixels overlaying the surface must be skipped (0;0;0) or exposed (255;255;255). All bitmaps represented in the article are inverted in color for better visualization. In this way, dimers were accurately positioned, the pit diameter in pixels corresponded to 100 nm, and the gap size was 12 nm. The FIB at 30 kV and 7 pA was set to etch patterns with 100 nm depth; the FIB etching depth is calibrated to the silicon etching rate. The glass etching rate is slower than it is for silicon, and it produces a reasonably correct depth of the pit (shallower than 80 nm).

Scan distortion of FIB must be considered when a few hundred nanometer dimer pits are planned to overlay a 20 × 24 μm^2^ area. At a larger FIB magnification, the precision of the beam positioning is higher. Lowering magnification, and thus the imaging/patterning larger area, the precision drops due to having a less precise beam deflection control and overall drop in scan resolution. Therefore, the only way to achieve high-resolution is by writing the patterns at a larger magnification (i.e., in a smaller area), then moving the stage to the next patterning place.

In order to cover the full spot size of the Raman laser, the pattern had to overlay a large area (at least 20 × 20 μm^2^). That is why FIB fabrication was automated by the Thermo Scientific iFast Developer’s Kit Professional software. The automation algorithm can be seen in the Supporting Information Figure S1 and the script itself, as Script file S3. The loop goes in the raster movement, and after each stage movement, a pattern file (corresponding to a 4 × 4 μm^2^ area consisting of 100 dimers) is fabricated by the FIB. In the case of randomly positioned pattern fabrication, the automation loop is supplemented with access to the bitmap folder (consisting of 30 different bitmaps), and in each subsequent loop, bitmaps are sequentially changed for patterning. The algorithm can be transferred to other dual-beam microscope control programs, and it can be easily modified for various needs.

**Figure 6:**
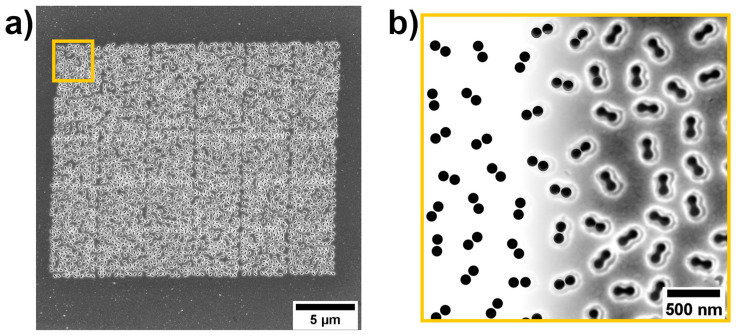
SERS substrate fabricated with FIB. (**a**) SEM image of full-patterned area of randomly positioned dimer system, and (**b**) corresponding zoomed-in yellow area with bitmap overlay onto a real high-resolution SEM image of fabricated structures.

Overall, the result of automation is a high-resolution nanostructure fabrication over a large area on a glass/Ag substrate from small, unique patterns (Figure 6a). The FIB-etched pits are slightly smaller than they were created in the bitmap (a SEM image with etched pits and a bitmap overlay can be seen in Figure 6b). Around the pits, a lighter area (wall) has been observed through the SEM images, and this indicates the redeposition around the etched areas.

**Figure 7:**
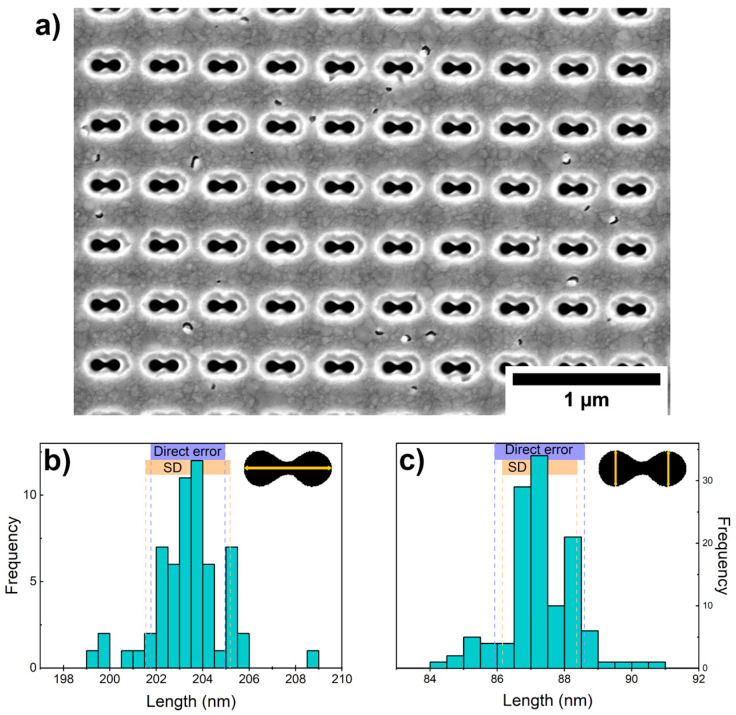
Size characterization of FIB-fabricated dimers. (**a**) SEM image of a periodically positioned dimer system; (**b**) Dumbbell-shaped pits’ horizontal length distribution and (**c**) vertical diameter distribution.

The accuracy of the etched structures was studied by analyzing SEM images using ImageJ software. The pits (black areas) were detected as “particles”, and three dimensions of each dumbbell-shaped “particle” were measured; in all, there were two maximum vertical diameters and a maximum horizontal length, shown in Figure 7a. The mean value of the normal distribution for the maximum horizontal length is 203.37 nm (Figure 7b), and the mean vertical diameter is 87.26 nm (Figure 7c), which corresponds to the observation in Figure 6b. However, the standard deviation in both measurements is close to the pixel size of the measurement (1 px = 1.36 nm), indicating high accuracy for FIB-fabricated structures.

### 3.3. Fabricated SERS Substrates

**Figure 8:**
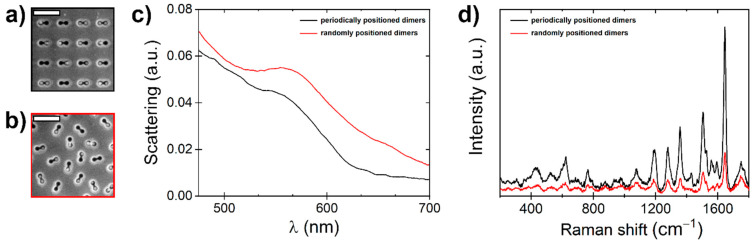
Characterization of dimers fabricated with FIB and filled with Au NPs. SEM images of (**a**) periodically positioned dimers and (**b**) randomly positioned dimers with a 500 nm scale bar. (**c**) Dark-field spectra of substrates and (**d**) 10^−5^ M RhB Raman spectra on fabricated substrates.

The 60 nm diameter gold NPs from the colloidal solution were filled into the pits using the capillary force-assisted deposition technique. SEM imaging showed three possibilities of gold nanoparticle positioning in the dimers (Figure 8a,b). Firstly, fully filled with two NPs; secondly, partially filled with one nanoparticle; and thirdly, completely empty dimer pits. Although the filling of NPs should be improved, the fabricated structures already showed potential properties for SERS.

In Figure 8c the dark-field (DF) spectra exhibit a shoulder around a wavelength of 550 nm, representing the anticipated peak in scattering efficiency for individual Au NPs with a 60 nm diameter [36]. In order to observe the coupling effects in Au nanoparticle dimers with a similar size, the interparticle gap needs to be reduced below approximately 20 nm [37]. This can potentially be achieved by tuning the template shape or nanoparticle size. The gap size is controlled by the dumbbell-shaped pit and nanoparticle geometry. Ideally, the nanoparticle and dumbbell lobe radii must be the same to maximize the adhesion force. In this case, the gap can be calculated by subtracting the nanoparticle diameter from the distance between lobe centers. The deposition of nanoparticles in pits without aggregation is achieved by balancing the capillary forces, adhesion, evaporation of wetting film, particle flow towards the meniscus area, and the withdrawal rate. These aspects are addressed in a separate study [34].

A 1 μL of 10^−5^ M RhB solution was drop casted on a 5 × 7 mm^2^ area. We estimate that there are 36 × 10^12^ molecules on the surface, and considering there is a 35 × 10^12^ nm^2^ area of the droplet, this results in approximately 1 mol/nm^2^. Therefore, each dimer (203 × 87 nm^2^) has ~17,000 adsorbed molecules, which were dried onto fabricated SERS substrates and measured with Raman spectroscopy. The dimers showed increased scattering around the wavelength of 550 nm; therefore, the green laser (532 nm) was chosen. In Figure 8d, it can be seen that periodically positioned dimers enhanced the signal more than those that were randomly positioned.

Both fabricated structures enhanced the Raman signal of 10^−5^ M RhB solution and made it detectable. The enhancement factor (*EF*) was calculated according to Equation (1), which is as follows [38,39]:

(1) $$EF=\frac{I_{SERS}/C_{SERS}}{I_{RS}/C_{RS}}$$

where *I_SERS_* is the Raman intensity and *C_SERS_* is the concentration of RhB used in SERS measurements; *I_RS_* is the Raman intensity and *C_RS_* is the concentration of RhB used in the Raman measurement (in this study, RhB powder). The calculated EF from the RhB 1644 cm^−1^ peak was 7.5 × 10^4^ on the glass surface, with randomly positioned dimer pits filled with gold NPs, and 2.9 × 10^5^ on the glass surface, with periodically positioned dimer pits filled with gold NPs.

## 4. Discussion

In this research, FIB was introduced as a proof of concept of a SERS substrate production method for high-resolution and large-area nanostructure fabrication. FIB was used as an indirect lithography, and the surface was prepared for filling the active nanostructures afterward. It can, however, be a direct lithography method as well, by only considering the ion–material interactions and redeposition. Additionally, it can be used as direct lithography to create high-resolution masks for different lithography methods, if the structure has proven its capability in the concept it was created for.

Large-area fabrication from small, unique patterns comes with a stitching problem. That is an issue that can be improved by modifying the algorithm. In Figure 6a, it can be seen that random dumbbell-shaped patterns are well-stitched vertically, but they have a skipped part by moving the stage horizontally. One way to improve this is by decreasing the stage movement step in the horizontal axis; the other way is to modify the pattern. A more advanced improvement would be obtained by adding image recognition in the algorithm to arrange the position of the already completed pattern, and the next one.

The deposition was conducted with capillary force assistance, and surface wetting plays a crucial role in this. We believe that post-processing (plasma cleaning and/or sonication) of the surface could improve its wettability, thus contributing to better gold nanoparticle deposition. Moreover, the deposition process could be optimized by adjusting parameters (the pulling rate, orientation of the substrate, temperature, concentration of the colloid, etc.) with respect to the fabricated surface. There might also be other routes to control nanoparticles on the surface, such as in situ synthesis of patterned gold nanostructures with a polymer brush [40] or solid-state dewetting of thin gold film [41], which could be combined with FIB-fabricated pits. However, as it is shown, the first tests on creating a well-controlled SERS substrate are promising.

The size distribution of NPs critically impacts their gaps on a prepatterned surface. Monodisperse NPs, with a narrow size distribution, are needed, as they enable precise control over interparticle gaps, creating uniform “hot spots” that are necessary for reproducible SERS signals. However, when particle sizes vary, as it can be seen in our study (Figure S2), these gaps become inconsistent and uncontrollable. Despite this drawback, asymmetric nanoparticle pairs with different sizes can also be practical, as they produce higher SERS intensities by creating a larger “hot spot” volume [42].

We have shown that depositing NPs into FIB-fabricated pits on the glass secures them in specific positions. We aimed to create a physical gap between NPs, and the FIB resolution shows promising results to ensure control over the distance between nanostructures. As already mentioned, the accuracy of the position can be ensured by physical methods, but the accuracy of mutual distances is ensured by chemical methods. A study on the combination of both methods was performed by Alexander et al. [23]. The highly controlled substrates were made to study the hot-spot and dimer orientation effect on SERS. Although the chemically created gap between the NPs is more precise, it is also more sensitive to the environment, and, in some cases, to radiation. Physically created gaps are more stable and probably can be used more than once for SERS purposes, after removing all organic matter from the surface. Therefore, we see the need to further study the capability of FIB to more precisely control the gaps between NPs.

Even though the pits were not completely filled with NPs, the substrates demonstrated an EF of 10^4^ and 10^5^ in SERS measurement. Periodically positioned dimers have a larger EF than randomly positioned ones, indicating that EF is highly dependent on dimer orientation and laser polarization matching [23]. Therefore, the adjustment of the laser and substrate is needed before every SERS measurement. Randomly positioned dimers do not require laser alignment, but the randomness is not easily repeatable.

Finally, FIB fabrication provides greater control over the nanostructure positions and flexibility due to the substrate material selection.

## 5. Conclusions

The high-resolution Phoenix Ion Column is suitable for the fabrication of a 100 nm structure when FIB is operated at 30 kV and the beam current is 90 pA or less. Working with a dual-beam microscope, Helios 5 UX, two parallel-etched ~ 40 nm wide lines (FIB, 30 kV, 7 pA) can be distinguished when the distance between the lines is 70 nm.

It is possible to etch any design SERS surface by creating a target file of the designed pattern. In order to minimize FIB scan distortions, the pattern is fabricated in an optimally sized area. Large-area fabrication is provided by automation, and the algorithm presented in the article should work on SEM-FIB microscopes that are similar to the Helios 5 UX. Therefore, the method is suitable for repeatable substrate fabrication.

The positions of the etched pattern structures correspond well to the positions of the design, but their sizes differ due to material redeposition during FIB exposure. Nevertheless, the FIB bitmap patterning with Helios 5 UX is highly accurate for high-resolution structure fabrication. The FIB (30 kV, 7 pA)-fabricated structure size distribution standard deviation is 1.53 nm in the horizontal structure dimension, and 1.07 nm in the vertical dimension (the analyzed image pixel size is 1.36 nm).

It has been demonstrated that FIB-etched dumbbell-shaped pits in glass can be filled with gold NPs by capillary force-assisted deposition. Therefore, the surface shows a proof of concept as a SERS substrate by detecting Rhodamine B and achieving an enhancement factor of 10^5^. The presented method shows a potential pathway for optical fiber functionalization.

Repeatable and adjustable SERS surfaces, such as well-controlled FIB-fabricated surfaces, help to continue the studies of SERS mechanisms. Therefore, the introduced method has the following two parallel future pathways: sensor development and a fundamental understanding of SERS.

## Data Availability

The original contributions presented in the study are included in the article and Supplementary Materials. Further inquiries can be directed to the corresponding author.

## Supplementary Materials

The following supporting information can be downloaded at: https://www.mdpi.com/article/10.3390/nano14221784/s1, Figure S1: Schematic automation cycle for periodical positioned dimer system fabrication, using iFast Developers Kit software with SEM Helios 5 UX; Figure S2: Gold nanoparticle size distribution, black curve represents normal distribution; Script file S3: The script of the automation cycle for periodic dimer system fabrication with FIB.
