# Supplementary figures and images for: Fabrication of Large-Area High-Resolution Templates by Focused Ion Beam Combined with Colloidal Nanoparticle Dimer Deposition for SERS Substrates
Source: Nanomaterials (Basel). 2024 Nov 6;14(22):1784. doi: 10.3390/nano14221784 (PMC11597278; doi:10.3390/nano14221784)

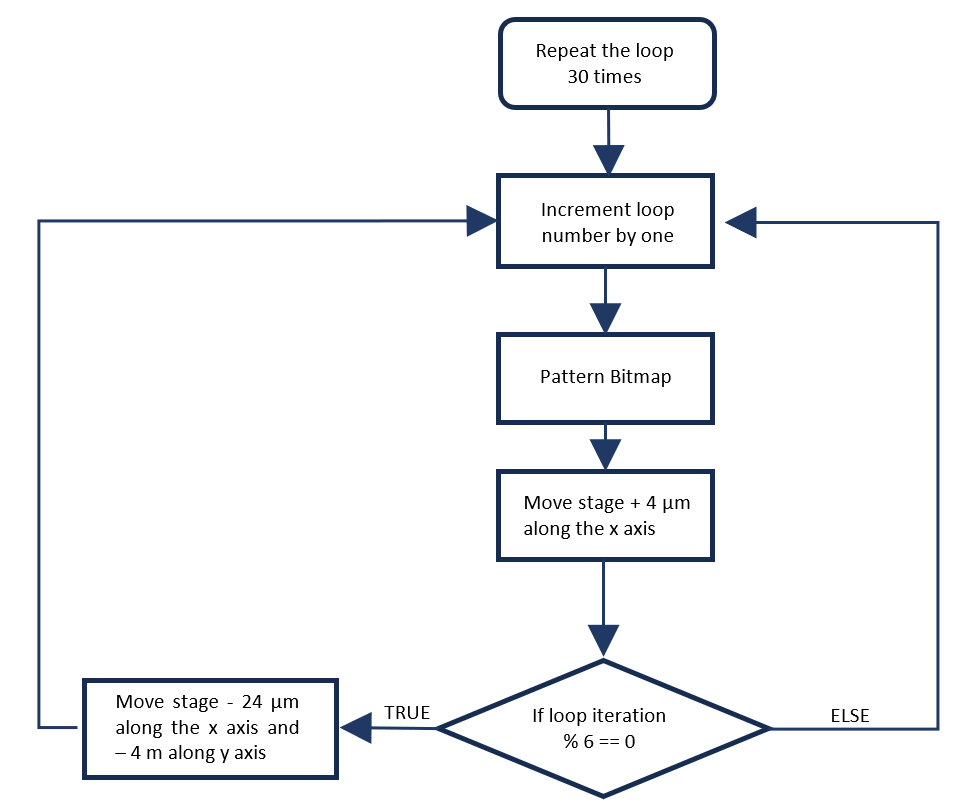

Supplement: Supplementary file 1 [file nanomaterials-14-01784-s001.zip › Figure S1. Schematic automation cycle for periodical positioned dimer system fabrication, using iFast Devel-opers Kit software with SEM Helios 5 UX.PNG]

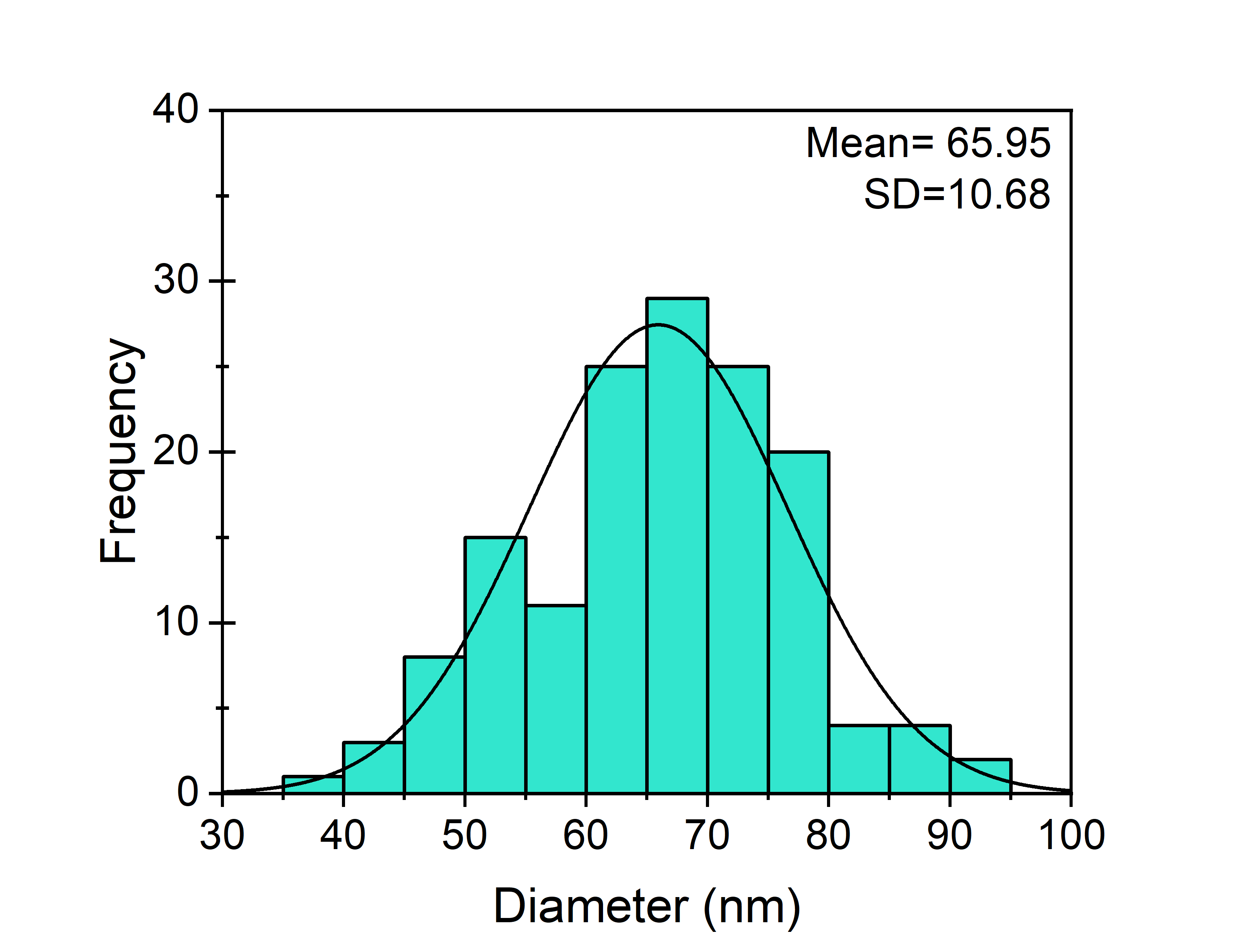

Supplement: Supplementary file 1 [file nanomaterials-14-01784-s001.zip › Figure S2. Gold nanoparticle size distribution, black curve represents normal distribution.png]
